# The Hidden Crisis: Understanding Potentially Morally Injurious Events Experienced by Healthcare Providers during COVID-19 in Canada

**DOI:** 10.3390/ijerph20064813

**Published:** 2023-03-09

**Authors:** Kim Ritchie, Andrea M. D’Alessandro-Lowe, Andrea Brown, Heather Millman, Mina Pichtikova, Yuanxin Xue, Maxwell Altman, Isaac Beech, Mauda Karram, Fardous Hosseiny, Sara Rodrigues, Charlene O’Connor, Hugo Schielke, Ann Malain, Randi E. McCabe, Alexandra Heber, Ruth A. Lanius, Margaret C. McKinnon

**Affiliations:** 1 Trent/Fleming School of Nursing, Trent University, Peterborough, ON K9L 0G2, Canada; 2 Department of Psychiatry and Behavioural Neurosciences, McMaster University, Hamilton, ON L9C 0E3, Canada; 3 Department of Psychology Neuroscience and Behaviour, McMaster University, Hamilton, ON L8S 4L8, Canada; 4 Department of Applied Psychology and Human Development, University of Toronto, Toronto, ON M5S 1V6, Canada; 5 Temerty Faculty of Medicine, University of Toronto, Toronto, ON M5S 1A8, Canada; 6 Homewood Research Institute, Guelph, ON N1E 6K9, Canada; 7 Atlas Institute of Veterans and Families, Ottawa, ON K1Z 7K4, Canada; 8 Institute of Mental Health Research at the Royal, University of Ottawa, Ottawa, ON K1Z 7K4, Canada; 9 Homewood Health Centre, Guelph, ON N1E 6K9, Canada; 10 St. Joseph’s Healthcare Hamilton, Hamilton, ON L8N 4A6, Canada; 11 Veteran’s Affairs Canada, Ottawa, ON K1H 1A1, Canada; 12 Lawson Health Research Institute, University of Western Ontario, London, ON N6A 3K7, Canada

**Keywords:** potentially morally injurious events, moral injury, healthcare providers, COVID-19

## Abstract

Background: Healthcare providers (HCPs) may be at elevated risk for moral injury due to increased exposure to potentially morally injurious events (PMIEs) throughout the COVID-19 pandemic. Identifying PMIEs experienced during the COVID-19 pandemic is a critical first step for understanding moral injury in HCPs. Accordingly, the purpose of the present study was to gain a deeper understanding of the work-related PMIEs experienced by HCPs in Canada during the pandemic. Methods: Canadian HCPs completed an online survey between February and December 2021 about mental health and functioning, including demographics and the Moral Injury Outcome Scale (MIOS). We conducted a qualitative thematic analysis of PMIEs described extemporaneously by HCPs in the open-text field of the MIOS. Results: One-hundred and twenty-four (*N* = 124) HCPs were included in analysis. Eight PMIE-related themes were identified, comprising patients dying alone; provision of futile care; professional opinion being ignored; witnessing patient harm; bullying, violence and divided opinions; resources and personal protective equipment; increased workload and decreased staffing; and conflicting values. Conclusions: Understanding broad categories of PMIES experienced by Canadian HCPs during the COVID-19 pandemic provides an opportunity to enhance cultural competency surrounding their experiences which will aid the development of targeted prevention and intervention approaches.

## 1. Introduction

Healthcare providers (HCPs) appear at elevated risk for moral injury during the COVID-19 pandemic due to the possibility of increased exposure to potentially morally injurious events (PMIEs). PMIEs are events involving: (i) self-perpetrated acts of commission or omission, where an individual transgresses their deeply held moral or ethical values by acting or failing to act; or (ii) witnessing acts of commission or omission by others, in some cases by a trusted authority, which violate one’s moral or ethical values [1,2]. Moral injury has been defined as a multidimensional outcome that some individuals experience post-PMIE exposure, characterized by profound moral emotions (e.g., guilt, shame, anger), symptoms of depression and anxiety, and an altered view of self and others [1,2]. Limited empirical evidence exists on moral injury and PMIEs in the healthcare context, especially relative to moral distress: the psychological distress that arises when a HCP is prevented from acting in line with their knowledge of an ethical course of action [3]. Moral distress may be expressed as feelings of frustration and anger, and of feeling belittled, unintelligent, or isolated [4] and has been associated with a range of negative mental health outcomes, including depression, anxiety, emotional detachment, guilt, and hopelessness [5]. It remains unclear to what extent moral distress and moral injury overlap. In their heuristic continuum of morally relevant life experiences and associated outcomes, Litz, and Kerig [6] posited that, as opposed to moral distress, moral injury may give rise to a heightened impact that extends across psychological, social, and spiritual domains. Here, moral injury is described as having a more holistic impact on individuals, giving rise, in some cases, to symptoms of post-traumatic stress disorder (PTSD) and related affective conditions, such as major depressive disorder [6].

The COVID-19 pandemic has sparked increasing interest in moral injury and PMIEs in healthcare. In a recent scoping review on moral distress and moral injury in HCPs during the COVID-19 pandemic, Riedel and colleagues [7] highlighted the blurred lines between the types of events that give rise to moral distress and PMIEs in the pandemic context by referring to events associated with moral distress or moral injury together as “moral stressors”. Here, moral stressors among HCPs during the pandemic include issues at the level of patient care (e.g., conflict between patient care and HCPs’ safety, witnessing inadequate care delivery), interpersonal relationships (e.g., working with HCPs who lacked knowledge of critical care, physician-nurse issues around treatment planning), and organizations (e.g., conflicts when distributing scare resources, lack of financial support, time, staff, and personal protective equipment) [7]. Relatedly, Xue and colleagues [8] conducted a scoping review to characterize extant knowledge surrounding circumstances related to moral distress and moral injury among HCPs worldwide during the COVID-19 pandemic. The results of this global scoping review revealed that potentially morally distressing or morally injurious events among HCPs worldwide during the pandemic included: (i) risk of contracting or transmitting the coronavirus; (ii) inability to work on the frontlines; (iii) provision of sub-optimal care; (iv) care prioritization and resource allocation; (v) perceived lack of support and unfair treatment by organization; and (vi) stigma, discrimination and abuse [8]. Despite a lack of consensus on the terms used to describe morally distressing or morally injurious events, collectively these reviews point towards widespread exposure to moral stressors, including PMIEs, among HCPs during the COVID-19 pandemic.

Understanding PMIEs experienced by HCPs during COVID-19 is critical given the elevated risk for moral injury and poor mental health outcomes associated with exposures. Emerging cross-sectional research reveals an association between PMIE-exposure and increased risk for deleterious mental health outcomes including the onset of symptoms of burnout, anxiety, depression, and PTSD, sleep difficulties, functional impairments, and suicidal ideation among HCPs [9]. Borges and colleagues [10] found that HCPs who experienced a PMIE had poorer psychosocial functioning compared to HCPs who were not exposed to PMIEs. Moreover, those HCPs who were exposed to a PMIE showed little relative improvement in functioning over a 10-month period [10]. Notably, Xue et al.’s [8] scoping review of circumstances related to moral distress and moral injury during the pandemic returned only four empirical investigations in Canada. As the impact of the pandemic varied greatly between nations given different waves, resources and crisis responses, understanding the types of PMIEs endured by Canadian HCPs remains a critical gap in the literature. Accordingly, the current study aimed to identify a common set of PMIEs encountered by Canadian HCPs throughout the pandemic that may also have long-term mental health consequences.

## 2. Materials and Methods

This study is part of a broader investigation of HCPs’ mental health and experiences during the COVID-19 pandemic and was approved by the Hamilton Integrated Research Ethics Board (#12667). Canadian HCPs were recruited to participate via social media, emails from consenting hospitals and healthcare organizations, and Participaid ads. Eligibility criteria specified that participants be at least 18 years of age, reside in Canada, speak and read English, and have contributed to patient care (e.g., frontline, support, administration) during the COVID-19 pandemic. Interested participants accessed the survey on Research Electronic Data Capture (REDCap) software [11,12]. The survey included a demographic form (e.g., age, sex, gender, occupation) and the Moral Injury Outcome Scale (MIOS) [2]. Data collection remains ongoing. The collected data were frozen and extracted in January 2022 for this analysis.

### 2.1. PMIEs during the COVID-19 Pandemic

The MIOS [2] is a novel self-report research tool developed within the military and Veteran population to assess moral injury as a multidimensional outcome, which remains to be validated among HCP [2]. The MIOS provides participants with a definition of distressing events consistent with current understandings of PMIEs and asks participants to indicate if they have ever experienced such an event. Participants who responded that they had experienced a PMIE are then asked additional questions about the event, including the year it occurred. Participants who report that they have not experienced a PMIE(s) are instructed to complete the instrument while keeping their most distressing event in mind. The MIOS includes a series of Yes/No questions about the event and associated outcomes, and an open-field text box for participants to describe the event, if they choose. The MIOS also includes 14 statements for which participants rate their degree of agreement on a scale ranging from 0 (strongly disagree) to 4 (strongly agree), and an additional item scored from 0 (not at all hard) to 6 (extremely hard) to describe the degree of impairment they experience in taking care of themselves or being effective in important spheres of life (e.g., work, school, getting along) [2]. For the purpose of this study, only open-field text box responses were extracted and qualitatively analyzed from the MIOS.

### 2.2. Data Analysis

Six-hundred and twenty (*N* = 620) HCPs participated in the survey between February and December 2021. Two-hundred and forty-three (*N* = 243) HCPs offered a response in the MIOS open-field text box. Open-field text boxes were included in the present analysis if the participant indicated (i) that the described event was a work-related PMIE and (ii) occurred during the years of the COVID-19 pandemic (i.e., 2020 and 2021). Thus, data were excluded if the event was not identified by the participant as a PMIE, was not related to work or occupation, or occurred prior the pandemic (i.e., prior to 2020).

Raw data were exported to Excel software for qualitative analysis. Qualitative thematic analysis followed Braun and Clarke’s [13] procedure of an inductive approach to identify and describe HCPs’ experiences. Conceptually, the analysis was informed by current definitions and understandings of moral injury as resulting from the actions or inactions of an individual, or witnessing actions of another, including betrayal by a trusted authority [1,2]. Two independent coders first familiarized themselves with the data and noted initial codes (K.R., A.M.D-L). Codes were collated into preliminary themes and were then reviewed to arrive at a thematic map of the data. Finally, the themes were refined and defined to arrive at a complete description of these data.

## 3. Results

The final dataset for the present study included 124 open-field text box responses. Demographic data for these participants are displayed in Table 1. Eight themes were identified from these data, reflecting HCPs’ common experiences of PMIEs during the COVID-19 pandemic: (1) patients dying alone, (2) provision of futile care, (3) professional opinions being ignored, (4) witnessing patient harm, (5) bullying, violence and divided opinions, (6) resources and personal protective equipment, (7) increased workload and decreased staffing, and (8) conflicting values.

### 3.1. Patients Dying Alone

Throughout the pandemic, and particularly during lockdown periods, policies were enacted to restrict visitors from hospitals. In many cases, these policies were applied unilaterally and were experienced as PMIEs for some HCPs. For example, a respiratory therapist wrote “[People] dying alone on several occasions despite no valid reason for policy.” (P345HCW). Although intended to protect patients and staff from infection, many participants perceived no-visitor policies to be a violation of their moral value of having family members present at end of life. Moreover, not granting exceptions to allow families to attend end of life was perceived as inadequate care, thus violating HCPs’ value of providing a peaceful and dignified end-of-life experience. For example, a physician wrote: “Patient was very sick/potentially dying with COVID-19 and family was not allowed to visit due to hospital policies.” (P60HCW). Witnessing patients die alone without family was particularly difficult when involving infants, and when HCPs were left as the only individual to comfort the patient. Here, a respiratory therapist shared that they “[h]ad to hold an infant while he passed away because hospital COVID rules would not allow his mother in to hold and comfort him when he died.” (P182HCW).

### 3.2. Provision of Futile Care

Many HCPs described the provision of perceived futile care as a PMIE. The majority of these situations were reported by respiratory therapists and nurses and often referenced critical care units where decisions were made to place patients on ventilators, even when they were not expected to survive. HCPs reported that decisions around invasive procedures, such as intubation and resuscitation were made even when they contravened the patients’ expressed wishes to either receive or not receive such invasive care:

“Present when a COVID 19 patient was talked out of an intubation by a physician she has previously indicated she wanted. It was questionable at the time if she had mental capacity to make that decision. That patient later died. Her family was unable to be there as she passed because of COVID restrictions so I held her hand while she passed.”

(respiratory therapist, P423HCW).

“ICU and ER physicians directed resuscitation on a patient who did not wish to be resuscitated. Distressing to staff as we all knew that this event was leading to a poor outcome anyways. This patient was denied a peaceful/dignified death. Aggressive resuscitation attempts happen often, staff (nurses, RT’s) follow orders to perform these tasks knowing that our efforts are futile.”

(respiratory therapist, P247HCW).

Decisions made by both members of the healthcare team and patients’ families to initiate or withhold life-prolonging treatment that transgressed the patient’s wishes were regarded as PMIEs. Feeling as though one did not advocate enough on the patient’s behalf was an added layer of distress when required to provide futile care. One nurse’s account read: “ICU patient. Didn’t advocate strongly enough for her. Family and medical staff went against her expressed wishes, and we tortured her on the ventilator for 3 months. She rotted in the bed until she died.” (nurse, P475HW). In these situations, HCPs reported feelings of failure when the patient was ventilated against their wishes and felt responsible for “not advocating strongly enough.” Acting in a way that contravened a patient’s wishes transgressed HCPs’ moral obligations to provide adequate, dignified, patient-centred care. HCPs perceived they had a responsibility to uphold the patient’s agency and represent the patient when they were too ill or no longer capable to express their wishes. One nurse described this as on ongoing issue that is exacerbated by a lack of organizational support:

“It’s been more of an ongoing issue. More and more the doctors I work with pass the buck so to speak as our patients are put through so much unnecessary suffering. On top of that, not having management support or a true ethics committee has put the pressure on us, the nurses to have conversations with families about what’s really happening with their loved ones. Also having families who want their loved ones being a full code [even] though the attempt would be futile and I’m causing suffering and pain to this person.”

(nurse, P98HCW).

### 3.3. Professional Opinions Being Ignored

Participants reported having their professional opinions and recommendations dismissed or ignored by team members and leadership as PMIEs, especially when a patient suffered harm or death as a consequence. A social worker explained: “Even though I did my job to the best of my ability, the decisions were trumped by management and completely went against what I know to be best, and what would have normally been viewed as important, no longer mattered.” (P472HCW). Similarly, a respiratory therapist offered the following example:

“Advocated for patient to receive treatment and interventions when early warning signs of patients’ deterioration were present. My advocacy and treatment suggestions were deferred until it became an emergency situation whereby the patient’s well being and prognosis were compromised.”

(P218HCW).

A common element in these descriptions is that patient harm could have been prevented had HCPs’ recommendations been acknowledged and followed. This was the case for a respiratory therapist who said, “I spent 2 h trying to get help for this patient and watched him go into cardiac arrest and die. The entire situation could have been avoided.” (P353HCW). These comments capture the struggle of frontline HCPs to be heard and the consequences of being unheard by leadership and other members of the healthcare team. When prevented from providing adequate care and the patient suffers harm, HCPs described lingering feelings of failure. Describing an experience of professional opinion being ignored and a patient consequently suffering harm, a respiratory therapist wrote:

“Patient deteriorating throughout dayshift, hospitalist ordered 3 lead sleep study (apnea-link) rather than follow RT advice to trial Bipap. Patient continued to have decreased LOC [loss of consciousness] (or sleeping?) overnight. Ended up requiring intubation and acute interventions.”

(P170HCW).

HCPs relatedly reported PMIEs involving patients who refused vaccination or medical advice while in the hospital and then suffered harm or death related to COVID-19. As one nurse said:

“Patient refused to get vaccine or vaccinate his elderly mother and contracted COVID. Patient refused to listen to writer or follow direction or medical advice with regard to self-proning in hospital, ended up needing to be intubated and on life support as a result. Writer [I] assisted with intubation after patient finished calling his loved ones to say goodbye in front of entire health care team involved in his care that he refused to listen to. It was too late.”

(nurse, P529HCW).

### 3.4. Witnessing Patient Harm

HCPs reported PMIEs that involved witnessing colleagues failing to provide proper patient care. PMIEs include participants both being present and required to participate in care they disagreed with or believed should have been handled differently but not having authority or ability to change the outcome. For example, a respiratory therapist wrote: “I assisted at a bedside tracheostomy that went very badly, resulting in the patient almost going into cardiac arrest, and the surgeon should have aborted the procedure when his resident pointed out significant risks of proceeding.” (P56HCW). Another respiratory therapist described witnessing patient harm due to the inactions of others: “I was paged to do a blood gas on a patient and found the patient pulseless, leading to me calling a code blue. I don’t think the patient was triaged appropriately, and never should have been left alone in an isolation room in emergency in that state.” (P178HCW).

Several participants also commented on situations where the actions of new or less experienced colleagues negatively impacted patient care. HCPs felt responsible for patient harm occurring as a result of the actions or inactions of those whom they were supervising: “Junior therapist did not ask for help when needed which affected patient care. I was working at the time and felt responsible to allow the potential harm to take place.” (respiratory therapist, P222HCW). Relatedly, a medical physician wrote:

“Resident doctors are first line of care for patients particularly during COVID-19. Occasionally, we encounter residents who did not want to see or refuse to see COVID-19 patients, particularly at night when there are no consultant physicians around. Sometimes we have to advocate strongly for patients to receive the care they need.”

(P516HCW).

Witnessing patients being treated poorly or neglected by colleagues was similarly reported as a PMIE by HCPs. For example, a Dietician described feeling helpless when witnessing patient harm in a long-term care home: “Witnessing resident neglect during COVID outbreaks in the nursing home, including rapid decline and death due to this neglect. And not being to do anything about it, prevent it or help people.” (P175HCW). Another nurse described feeling helpless when witnessing patient harm from a COVID-19 outbreak: “During the COVID outbreak at my work—I felt helpless for the residents that died and that I let their family down.” (P563HCW). In addition to the distress from witnessing patient harm, these comments also bring attention to HCPs perceived inability to change the harmful situation and feelings of helplessness. 

### 3.5. Bullying, Violence, and Divided Opinions

Multiple instances of workplace bullying, harassment, or acting in an uncaring manner toward colleagues or leadership were described as PMIEs and often left HCPs feeling incompetent and demoralized. HCPs reported that their employer acted in a way that was perceived as unethical or uncaring towards their employees: “I asked for support from my manager/director to help deal with burnout and was called over-the-top and mentally ill by senior management.” (occupational therapist, P359HCW). Describing a PMIE with a manager and its impact on staff retention, a nurse wrote: “New manager bullied myself and other teammates during the pandemic resulting in loss of staff who resigned due to the actions of the manager and inaction of senior leadership.” (P617HCW). Relatedly, another nurse shared of being personally harmed by the demoralizing actions of others:

“Not being able to understand a protocol despite asking the charge nurse who made me feel incompetent. That feeling made me feel demoralized, so I didn’t follow up and made errors. There was no patient harm but there was personal harm to myself.”

(P663HCW).

HCPs reported acts of violence by co-workers toward clients: “Co-worker sexually inappropriate with vulnerable, young, female clients. Was ultimately terminated after an extended investigation, [I] was accused of ‘being out to get him.’” (nurse, P278HCW). HCPs also reported divided opinions among health teams regarding extreme views about vaccines. A respiratory therapist said:

“I am having extreme difficulty with the extreme opinions exhibited about vaccine status amongst health care workers. Many providers believe that people who have not [been] vaccinated are not deserving of treatment. While I firmly believe in vaccination, I do not agree with this line of thinking and it often puts me at odds with my colleagues. This causes great moral distress for me. I have difficulty going to work and anxiety while I am there.”

(P413HCW).

### 3.6. Resources and Personal Protective Equipment (PPE)

Issues with resources and PPE limited patient care and were described as PMIEs during the pandemic. Several participants highlighted that PPE policies established to limit the risk of COVID-19 exposure for HCPs, although intended to protect HCPs from infection, actually made it more difficult, or completely prevented, a timely response to emergency situations. A respiratory therapist shared: “With COVID-19 protocols for PPE, I have seen delay in providing resuscitation immediately and in a timely way cause both mortality and morbidity when I feel strongly that outcome would have been/could have been different.” (P370HCW). Further, organizational decisions to preserve the short supply of PPE were at times perceived by HCPs to be unfair, putting some HCPs at greater risk of infection than others. For example: “[Organization] refused to provide PPE to nurses on MH [mental health] team [and instead] save it for ER nurses.” (social worker, P106HCW).

Some participants described patients sustaining harm from room setup or a lack of resources: “Code blue, COVID intubation in isolation room. Unable to communicate well with staff due to room setup. Watched patient arrest during intubation attempt and nothing done due to COVID precautions and lack of appropriate equipment.” (respiratory therapist, P220HCW).

### 3.7. Increased Workload and Decreased Staffing

HCPs described PMIEs when increased community transmission of the coronavirus drastically increased their workload, limiting the ability to provide quality care. A respiratory therapist explained being “Unable to complete a task as thoroughly as I would before because of being pulled to several patients at once and then the patient deteriorated.” (P288HCW). While workloads increased over the course of the pandemic, many care settings additionally experienced decreased levels of staffing. Policies restricted many staff from working if they had any symptoms of COVID-19 or had been in close contact with someone who had COVID-19. This meant that staff were responsible for caring for more patients than could be managed. As one nurse described:

“I am to oversee more patients than is safe for me to do so. This patient has been stable but began deteriorating and was noticed late. If I hadn’t been relying on my peers for part of an assessment, maybe I would’ve noticed something was wrong sooner. Maybe the outcome would’ve been different. Maybe I could’ve done something differently.”

(P630HCW).

HCPs working in outpatient and community healthcare facilities reported receiving calls from patients and their families desperately trying to find help for themselves or their loved ones. Many of these facilities and clinics were closed for periods of time or not operating at full capacity during the pandemic. Thus, participants reported being unable to offer patients and families usual care in the form of appointments or referrals to other community agencies. HCPs described PMIEs stemming from their inaction or being placed in situations where they had no choice but to turn patients and families away despite knowing that they needed help, or that care refusal could potentially cause them harm. An occupational therapist shared:

“Dealing with addictions programs closing because of the pandemic, had patients showing up at the clinic who I couldn’t help, nor let in the building. They wanted the help but were unable to access care. Many small moral injuries over time, no one specific event.”

(P263HCW).

### 3.8. Conflicting Values

Multiple participants described PMIES involving a discrepancy in values between themselves and management. HCPs perceived that management was placing less value on patient wellbeing and more value on saving costs and overall organizational effectiveness. A nurse shared:

“Covid patient in distress for most of day. No medication or inhalers would improve condition. Both RT and I could do nothing. All the while management forcing me to move this patient to another floor to make room for a surgical patient. This was end of second wave. The day was so chaotic and busy… that woman was in distress and didn’t get the care she deserved or needed.”

(P268HCW).

HCPs described being “forced” by system level issues to act in a way that contravened their value for prioritizing patients’ wellbeing. In some cases, this was perceived by HCPs as a disconnect between the values of management and those of the HCPs, resulting in a feeling of betrayal. For example, a nurse shared:

“A palliative patient in a pain crisis came to the ED requiring a drip of narcotics, which was planning for him to go home on the next day. [He was forced] to stay in the ED for the next 48–54 h because no other floor in the hospital would take him and accept his care. So he stayed on a stretcher in our noisy resuscitation room [for] 4 h.”

(P91HCW).

## 4. Discussion

This investigation provides an account of work-related PMIEs experienced by Canadian HCPs during the COVID-19 pandemic. Whereas in some examples HCPs reported witnessing the actions or inactions of colleagues as PMIEs (e.g., neglect of patients’ care needs; failure to prioritize patient care over other responsibilities; errors made by students or junior HCP), in other instances HCPs themselves felt responsible for contributing to a lowered standard of care delivery resulting in patient harm (e.g., feeling that one did not advocate enough; not acting fast enough due to PPE). Regardless of the perceived perpetrator of a PMIE, or whether a value was violated through acts of commission or omission, it would seem that a core moral value repeatedly transgressed for HCPs during COVID-19 was the ability to provide adequate patient care and to minimize patient harm. HCPs place immense value on prioritizing patient wellbeing, the alleviation of suffering, prevention of harm, and provision of choice and high-quality care [7,14]. Akram et al. [15] noted that the very nature of the relationship between the HCP and their patients’ conflicts with the utilitarian nature of healthcare delivery and may be a central component of moral injury in HCPs. Indeed, in the present study, PMIEs involving system and organization level policies and decisions sometimes forced HCPs to act in ways inconsistent with their duty to put patients’ needs first (e.g., having to enforce no-visitor policies). Some of these policies led to sub-standard patient care, including delays in providing emergency treatments, patient harm and, in some cases, death. At times throughout the pandemic, the provision of adequate individual patient care and the ability to minimize suffering came into conflict with well-intentioned public health policies that followed more utilitarian principles intended to slow the spread of infection, reduce the burden on the healthcare system, and protect the most vulnerable [15]. These novel guidelines to reduce the spread of infection, while serving the needs of the greater population, also created moral conflict within HCPs who perceived their core moral values to be transgressed.

Interestingly, themes related to the violation of adequate patient care and minimizing patient harm elucidated in the present study are consistent with hallmark examples of morally distressing healthcare situations occurring at the patient, team, and system levels (e.g., having to proceed with invasive care at family member’s requests; working with team members who lack appropriate skill or training; feeling unsupported by administration) [3]. Unresolved value conflicts may connect current conceptualizations of morally distressing events and PMIEs. Moral distress and moral injury involve a fundamental conflict between one’s beliefs or values concerning the right course of action in a given situation [16]. Previous research suggests that moral distress in healthcare is centered primarily on occupational phenomena caused by stressors that violate deeply held values associated with HCPs’ professional roles [3] or that result in feelings of being “trapped” by organizational constraints [16]. Although these situations occur primarily in occupational settings that violate professional values, moral injury is traditionally thought to occur within the context of situations that violate deeply held personal beliefs and values [2,16]. In a healthcare context, however, any distinction between professional and personal values appears ambiguous where professional values may become personal values for HCPs, related to a deep sense of fulfillment when caring for others and effectively carrying out one’s role. Thus, it would appear that central to the PMIEs described here, that unresolved value conflicts, both within the HCP (e.g., events violating their personal values; bullying and harassment) and between the HCP and the system in which they work (e.g., policies or procedures conflicting with the HCPs’ professional values). The relation and distinction between morally distressing events and PMIEs should be further investigated to better understand these concepts within healthcare.

The types of events reported by Canadian HCPs in the present study are in keeping with the literature on COVID-19-related PMIEs reported by HCPs globally. For example, in a study of 350 American HCPs surveyed between September and December 2020, about half of the participants reported feeling troubled by witnessing other’s immoral acts [9]. Here, witnessing harm or neglect to patients was associated with feelings of helplessness among these HCP [9]. In a related study, Liberati and colleagues [14] found evidence of potential moral injury among English HCPs who felt forced to provide sub-standard mental healthcare during the pandemic, including isolating vulnerable individuals to prevent infection or transmission and patient harm due to delays or changes in care delivery. These findings are in keeping with prior research indicating that being unable to provide adequate care may result in moral distress that is associated with guilt and burnout [16].

Notably, some PMIEs described by Canadian HCPs in the present study may overlap with other stressor classifications, including potentially psychologically traumatic events (PPTEs) [17]. PPTEs are events which may give rise to a diagnosis of PTSD, involving exposure to actual or threatened death, injury or sexual violence [17,18]. PPTEs have been found to increase the likelihood of symptoms of mental disorders, including PTSD, major depressive disorder and generalized anxiety disorder among nurses [19]. While the situational features of PMIEs and PPTEs may intersect (e.g., patient death as a result of inability to provide adequate care due to lack or resources), in some cases, these stressor classifications may be distinct (e.g., bullying and demoralization from management and coworkers that does not involve actual or threatened death, injury, or sexual violence). In order to support HCPs during and beyond the pandemic period, it will be important to acknowledge and address PMIEs that do not involve PPTE features (in addition to events with overlapping elements) as PMIEs have been associated not only with moral injury symptoms but with additional adverse psychological responses (e.g., symptoms of depression, anxiety and PTSD, suicidal ideation) [1,6]. For example, as HCPs in the current study reported PMIEs related to work-related interpersonal issues, including bullying and harassment that did not intersect with PPTEs, Canadian nurses previously reported similar interpersonal work conflicts and labeled these events the most impactful and distressing work event endured to date, including events that did not involve actual or threatened death, injury or sexual violence [20]. Here, future research is required to understand the impact of PMIEs, both independently and when combined with PPTEs so that adequate supports and resources may be developed.

### Strengths and Limitations

The present study adds to our understanding of moral injury in a healthcare context and can be used to generate patient, team, and organizational level prevention and intervention strategies to ward against moral injury. This study is not without limitations. The present study is limited in its cross-sectional design and does not reflect all PMIEs that occurred across Canada during the COVID-19 pandemic given an unrepresentative sample of Canadian HCPs. Furthermore, the findings of the present study are not generalizable to all Canadian HCPs as the sample was composed primarily of female-identifying respiratory therapists and nurses residing in Ontario. Future research should consider more in-depth first-hand accounts of COVID-19-related PMIEs from HCPs across Canada that are more representative of the country’s diverse occupational and demographic groups.

## 5. Conclusions

This investigation identified and described work-related PMIEs encountered by Canadian HCPs during the COVID-19 pandemic. HCPs reported a range of PMIEs during the pandemic that negatively impacted quality of care and patient outcomes. These data add to our understanding of moral injury in a healthcare context and can be used to inform prevention strategies at the patient, team, and organizational level of healthcare to address PMIE exposure and mitigate moral injury and its associated negative mental health outcomes.

## Figures and Tables

**Table 1:** Sample Demographics.

		Frequency	Percentage
Occupation			
	Medical Physician	10	8.06
	Nurse	32	25.81
	Occupational Therapist	10	8.06
	Respiratory Therapist	52	41.94
	Social Worker	<5	-
	Other *	17	13.71
Sex			
	Female	108	87.10
	Male	14	11.29
Age			
	20 to 29	19	15.45
	30 to 39	48	38.71
	40 to 49	28	22.58
	50 to 59	21	16.94
	60 to 69	7	5.65
Province			
	Alberta	12	9.68
	British Columbia	21	16.94
	Manitoba	5	4.03
	New Brunswick	<5	-
	Nova Scotia	5	4.03
	Ontario	72	58.06
	Prince Edward Island	<5	-
	Quebec	<5	-
	Saskatchewan	5	4.03

* Note: “Other” occupations included activation manager (1), administration (1), advanced care paramedic (1), dental hygienist (3), dietician (2), director of operations (1), nursing unit assistant (1), office manager (1), personal support worker (1), physiotherapist (1), psychotherapist (2), resident physician (1), speech language pathologist (1). Frequency counts less than 5 are collapsed for anonymity. Some participants did not provide responses to all demographic questions; as such their data is missing and the total frequency counts for those variables are less than 124.

## Data Availability

The data used in this study come from the McKinnon Trauma and Recovery Research Unit at McMaster University. All interested researchers may apply for access to these data through online application subject to review by the Data Access Committee, ethics approval, and signing of a data sharing agreement. Data are provided only once a data sharing agreement is in place between McMaster University (the custodian of the data) and the researchers’ institution. For more information about data access please contact https://www.thetraumaandrecoverylab.com/contact.
